# Comparative evaluation of platelet-rich fibrin and collagen membrane for guided bone regeneration in alveolar defects

**DOI:** 10.6026/973206300222779

**Published:** 2026-05-31

**Authors:** Nitish Kumar Kompalli, Shivani Mishra, Tamma Prathyusha Reddy, Isha Agrawal, Rajat Dabholkar, Sukanya Mishra

**Affiliations:** 1 Department of Dentistry, Kotilingam's Dental Clinic, Guntur Andhra Pradesh, India; 2 Department of Dentistry, Virendra Kumar Sakhlecha Government Medical College, Neemuch, Madhya Pradesh, India; 3 Department of Dentistry, Sri Ramachandra institute of higher education and research, Chennai, Tamil Nadu, India; 4 Department of Periodontics and Implantology, Manubhai Patel Dental College and Hospital, Vadodara, Gujarat, India; 5 Department of Prosthodontics and Crown & Bridge, Yogita Dental College and Hospital, Khed, Maharashtra, India; 6 Department of Periodontics, Kalinga Institute of Dental Sciences, Bhubaneswar, Odisha, India

**Keywords:** Platelet-rich fibrin (PRF), collagen membrane, guided bone regeneration, alveolar defect, bone augmentation

## Abstract

Alveolar bone defects following tooth loss, trauma, or pathology compromise implant placement and prosthetic rehabilitation, necessitating effective guided bone regeneration strategies. Therefore, it is of interest to compare the clinical and radiographic outcomes of platelet-rich fibrin (PRF) and resorbable collagen membranes in alveolar defect regeneration. Hence, forty patients were randomly allocated into PRF (n=20) and collagen membrane (n=20) groups, both combined with particulate bone graft, with evaluation of healing parameters and CBCT-based bone gain at baseline and 6 months. Both groups demonstrated significant bone regeneration, with slightly greater horizontal gain in the collagen group, while vertical gain and complication rates showed no statistically significant differences. PRF and collagen membranes exhibit comparable regenerative efficacy, with PRF offering additional advantages of autologous origin, cost-effectiveness, and enhanced soft tissue healing.

## Background:

Resorption of the alveolar ridge after loss of teeth is a ubiquitous biological event which greatly undermines the functional and esthetic restoration of edentulous sites. Immediately bone resorption begins following the extraction of teeth and it becomes high in the first three to six months and it is reported to lose up to 50% of the alveolar ridge width in the first year [1]. This dimensional decrease leaves the bone volume inadequate to allow the ideal positioning of endosseous dental implants and bone augmentation surgeries might be necessary before or alongside the implant surgeries[2].The level and pattern of alveolar resorption is very different in individuals and depends on factors such as the extraction method, socket morphology, local infection, systemic conditions and ridge preservation measures (present or absent)[3].Guided bone regeneration has proven to be one of the surest and most documented procedures to use in augmentation of underdeveloped alveolar ridges as well as in correcting peri-implant bone defects. The basic biological principle of guided bone regeneration entails the placement of a barrier membrane to physically isolate the space of a lesion to more slowly migrating osteoprogenitor cells, but not the faster migrating epithelial and connective tissue fibroblasts [4]. This space preservation and wound stabilization are coupled with this cell-occlusive activity of the membrane which ensures the environment where osteogenesis is not disturbed in the secure compartment [5]. Guided bone regeneration membranes can be classified into broad categories since they are either non-resorbable or resorbable. Non-resorbable membranes such as expanded polytetrafluoroethylene have good space creation and predictability, but need removal in a second surgery, which is more patient morbid and complex to treat [6]. The use of resorbable membranes has become widely popular because they did not involve the use of secondary surgery and showed similar clinical results in most indications. Of all the currently available resorbable membranes, the collagen-based membranes of bovine or porcine origin are considered the gold standard in resorbable membranes because of their strong biocompatibility, good handling properties, encouragement of fibroblast attachment and regulated biodegradation behavior [7]. Nevertheless, collagen membranes are linked to few restrictions such as high costs, tendency to cause immunogenic response when sensitized, chances of transmitting diseases through xenogeneic sources and inconsistent rates of degradation that leads to impairment of barrier properties before proper bone regeneration is achieved [8]. Such constraints have provoked curiosity in the development of autologous biological substitutes that may offer the benefit of barrier m embrane properties in addition to the delivery of endogenous growth factors and bioactive molecules which can supplement the process of regeneration. Platelet-rich fibrin which has been presented as second-generation platelet concentrate has gained much interest in this regards [9]. In contrast to its parent platelet-rich plasma, PRF is prepared by a simplified single-centrifugation procedure and not supplemented with anticoagulants or bovine thrombin to create a natural fibrin network that entraps platelets, leukocytes and circulating stem cells in its architecture [10].This fibrin network acts as a slow release delivery system of a selective combination of growth factors such as platelet-derived growth factor, transforming growth factor-0, vascular endothelial growth factor, insulin-like growth factor and epidermal growth factor, which are slowly released through the physiological remodeling of the fibrin matrix by a period of seven to fourteen days [11]. PRF has other biologicalproperties other than being a growth factor. The thick fibrin structure offers a three-dimensional structure into which the cells migrate and angiogenise and the leukocytes embedded on them help regulate the immune response and provide resistance to infections at the surgical site [12].Consolidated into membrane format, PRF may serve as a barrier membrane, which may confer the cell-occlusive effects necessary to regenerative pathway bioactivity in addition to the bioactivity of regenerative pathways [13]. Moreover, the PRF membranes are fully autologous, which excludes the issue of immunogenicity and disease transmission, as well as any ethical issues that might be related to xenogeneic materials and also provide major cost benefits since they are made of the patient own blood [14]. Although PRF has a theoretical benefit, clinical data comparing PRF membranes to traditional collagen membranes in guided bone regeneration are rather too limited and inconclusive. The application of PRF in socket preservation and ridge augmentation is also studied by several studies and the overall results are generally positive [15]. Nevertheless, there are still doubts about PRF membranes in terms of mechanical stability and lifespan, which decay faster than collagen membranes and might not deliver sufficient barrier activity during the most crucial initial stages of bone regeneration [16]. Compared to four to six month's degradation profile of cross-linked collagen membranes, the comparatively fast resorption of PRF, which usually takes two to four weeks to occur, has been suggested to have clinical implications that are yet to be fully explained [17]. Moreover, a majority of the available comparative research has examined extraction socket healing as opposed to the augmentation of pre-existing alveolar defects which pose a more difficult clinical case given that their vascularity has been compromised, defect size has been increased and regenerative capacity is diminished [18].Therefore, it is of interest to compare the clinical and radiographic outcomes of platelet-rich fibrin and collagen membranes in guided bone regeneration of alveolar defects.

## Materials and Methods:

## Ethical and design of the study:

This was a prospective, parallel-group, randomized controlled clinical trial that was carried out at the Department of Oral and Maxillofacial Surgery in March 2022-September 2023. The study was initiated with ethical approval of the Institutional Review Board being received beforehand. The trial was enrolled in a clinical trials registry and it was carried out in the spirit of the principles given in the Declaration of Helsinki. The informed consent was written and signed by all the participants after thorough explanation of the study protocol, expected results, possible risks and choice of alternative treatments.

## Sample size determination:

The calculation of the sample size was done according to the G*Power software (version 3.1.9.7) which was calculated on the basis of the initial data of pilot study with eight patients. A two-tailed independent samples t-test with an expected effect among horizontal bone g ain (primary outcome) of 0.65, a 0.05 alpha and a 0.80 statistical power resulted in the calculating of a minimum of 17 patients per group. Twenty patients were recruited in each of the groups as a result of the possibility of having a dropout rate of 15 percent thus making the sample forty in total.

## Patient selection:

Eligibility screening was done on patients who reported to the outpatient clinic with alveolar ridge defects, which demand bone augmentation before placing implants. On the randomization and group allocation, 3.4 will be used.

## Inclusion criteria:

[1] Age between 20 and 60 years

[2] Horizontal alveolar ridge deficiency not less than 3 mm (Seibert Class I or Class III defect)

[3] Three months have passed since tooth extraction.

[4] Sufficient soft tissue to close the wound and to close the wound primary.

[5] Excellent general health (ASA I or II).

[6] Good oral health (PI 0.5 and less).

[7] The desire to adhere to follow-up procedures.

## Exclusion criteria:

[1]Uncontrolled systemic illnesses (diabetes mellitus and HbA1c is more than 7, autoimmune diseases)

[2]Smokes cigarettes (> 10 cigarettes per day)

[3]Past history of bisphosphonate therapy or head and neck radiation.

[4]Chronic corticosteroids or immunosuppressant use.

[5]Hematological diseases or abnormalities in coagulation.

[6]Periodontal disease in neighboring teeth.

[7]Pregnancy or lactation

[8]Bovine or porcine collagen product allergies.

[9]Existence of acute infection at the area of surgery.

The random assignment of the patients to either of the two groups was performed using a computer-generated sequence of random numbers and was randomized (block randomization with block size four). Sequentially numbered, opaque and sealed envelopes were used to preserve allocation concealment and these were opened by the surgical assistant just prior to the surgery:

[1] GRP RF (n = 20): autologous PRF membrane -particulate xenogeneic bone graft guided bone regeneration.

[2] Group COL (n = 20): Bone regeneration on guided bone regeneration with resorbable collagen membrane (Bio-Gide®, Geistlich Pharma, Wolhusen, Switzerland) with the same particulate xenogeneic bone graft.

## PRF preparation:

In the case of patients assigned to PRF group, the venous blood was taken just before the operation. Anticoagulant-free 10-mL glass-coated vacationers of antecubital vein venous blood were used. Tubes were then centrifuged at 2700 rpm (or just under 400 g) at 12 minutes with a fixed angle table top centrifuge (Process for PRF, Nice, France). The resulting PRF clots were carefully removed off the tubes by using sterile forceps; they were separated off the red blood cell base and squeezed into membranes by use of a sterile PRF compression box. Membranes were moistened in the exudate which was gathered during compression until surgical fixation.

## Surgical protocol:

A single experienced surgeon carried out all surgical practices under local anesthesia (articaine 4 percent with 1:100,000 epinephrine). One hour before the operation, prophylactic treatment was used with amoxicillin 2 g. A mid-crestal incision was performed including vertical releasing incisions on the adjacent teeth and full-thickness mucoperiosteal flaps were raised on both the lingual and buccal sides. The alveolar defect was opened and severely debrided of all granulation tissue. A small round bur was used to make cortical perforations with sterile saline irrigation in order to encourage bleeding and allow migration of osteoprogenitor cells out of the marrow spaces. To fill the defect, 0.251 mm bovine bone mineral (Bio-Oss100) was hydrated with sterile saline and gently packed into the defect to the desired shape (deproteinized, Bio-Oss200) which was in the form of a granulometry with a diameter of 0.25-1 mm. In PRF group, the particulate graft was lined with two sheets of compressed PRF membranes that ranged at least 2-3 mm further than the edges of the defects on all sides. Under collagen group, a resorbable collagen membrane was cut to a size that was fit and placed over the defect that is being grafted with a similar overlap. The tacks were resorbable and put in place to stabilize membranes. At the bottom of the buccal flap, periosteal releasing incisions were done to ensure tension free primary closure. Horizontal mattress and interrupted suture repair with 4-0 polyglactin (Vicryl) suture material was used in wound closure.

## Post-operative protocol:

All the patients were provided with the same treatment in terms of postoperative medications:

[1] Amoxicillin 500mg three times per day in seven days.

[2] Ibuprofen 400mg three times/day during five days.

[3] Chlorhexidine digluconate 0.12% mouth rinse 2 times a day in two weeks.

The patients were advised to prevent mechanical disturbance of a surgical area, keep to a soft diet within two weeks and not to wear removable prosthesis over the area of augmentation at least in eight weeks. At 14 days postoperative, the sutures were removed.

## Clinical assessment:

Clinical assessment was done at post-operative days 7, 14 and 30 and at three and six months. The parameters recorded were as follows:

[1] Wound dehiscence: Exposure or wound breakdown of membrane at every visit

[2] Soft tissue healing index: Measured by modified Landry healing index (1-5: 1 very poor, 5 excellent) in days 7 and 14.

[3] Post-operative complications: Infection, swelling, excessive pain, membrane exposure, graft loss recorded on every visit.

[4] Ridge width: The width of the ridges at the crestal level measured clinically with a ridge mapping caliper at six months.

## Radiographic assessment:

At the baseline (just before surgery) and six months after surgery, Cone-beam computed tomography (CBCT) scans (Planmeca ProMax 3D, Planmeca Oy, Helsinki, Finland) were taken with the same exposure parameters (90 kV, 8 mA, voxel size 0.2 mm, field of view 8 x 8 cm). The analysis of CBCT images was done through specialized implant planning software (Romexis 6.0, Planmeca) by one blinded examiner who was not informed of the groups.

Radiographic measurements were then made at three standard cross-sectional points in each defect and they were:

[1] Horizontal bone width (HBW): It is measured 1 mm, 3 mm and 5 mm below the alveolar crest.

[2] Vertical bone height (VBH): This is a measurement between the most coronal point of the regenerated bone and an established reference plane (inferior border of the mandible or palatal plane at maxillary locations).

[3] Bone density: Measured by mean gray values in the augmented area.

Horizontal bone gain was determined by taking the difference between the horizontal width (six months and baseline). Vertical change in bone was also computed.

## Statistical analysis:

The SPSS version 26.0 (IBM Corp., Armonk, NY, USA) was used to conduct statistical analysis. The variables that were continuous were in mean plus SD. To determine the normality of data, the Shapiro-Wilk test was employed. Between groups differences in normally distributed data were tested using independent samples t-tests. The Mann-Whitney U test was used in the variables that are not normally distributed. Within-group comparisons were made in the basis and six months measurements using paired t-tests. The Fisher exact test was used to test the differences between categorical variables. Intra-examiner reliability regarding radiographic measurements was estimated by determining the intraclass correlation coefficient. The statistical significance was defined to be below p = 0.05

## Results:

Forty patients were enrolled and thirty-seven completed the six-month follow-up protocol. Three patients were excluded during the study: one from the PRF group due to postoperative infection requiring premature intervention and two from the collagen group due to loss to follow-up. The final analysis included 19 patients in the PRF group and 18 patients in the collagen group. The demographic and baseline characteristics of the study population are presented in Table 1 No statistically significant differences were observed between groups for any baseline parameter, confirming adequate randomization. Soft tissue healing was generally favorable in both groups. The modified Landry healing index scores at day 7 and day 14 are summarized alongside wound dehiscence and complication rates in Table 2 The PRF group demonstrated significantly higher healing index scores at day 7 compared to the collagen group (p = 0.028), indicating more favorable early soft tissue healing. By day 14, healing scores were comparable between groups. Wound dehiscence with membrane exposure occurred in one patient in the PRF group (5.3%) and three patients in the collagen group (16.7%), though this difference did not reach statistical significance (p = 0.334). One patient in the collagen group with membrane exposure subsequently developed localized graft infection requiring partial graft removal. The radiographic measurements at baseline and six months, along with calculated bone gain values, are presented in Table 3 Both groups demonstrated statistically significant horizontal and vertical bone gain compared to baseline (p < 0.001 for all within-group comparisons). The collagen group showed slightly greater mean horizontal bone gain at the crestal level (3.74 plusnm;1.18 mm vs. 3.21 plusnm;1.06 mm) and this difference approached but did not reach statistical significance (p = 0.154). At the 3 mm and 5 mm subcrestal levels, horizontal bone gain was comparable between groups with no significant differences. Vertical bone gain was similar in both groups with no statistically significant difference. Mean bone density values in the augmented area at six months were also comparable between groups. The intraclass correlation coefficient for intra-examiner reliability of radiographic measurements was 0.94 (95% CI: 0.91-0.97), indicating excellent measurement reproducibility. At the six-month reentry surgery, all 19 patients in the PRF group and 17 of 18 patients in the collagen group had sufficient bone volume for implant placement in the planned positions. One patient in the collagen group who experienced partial graft loss required additional grafting at the time of implant placement. The overall implant placement feasibility rate was 100% in the PRF group and 94.4% in the collagen group (p = 0.486).

## Discussion:

The current randomized controlled clinical trial was done to compare clinical and radiographic outcomes of platelet rich fibrin membranes and resorbable collagen membranes in guided bone regeneration of alveolar defects. The findings showed that both types of membranes both attained substantial and clinically significant bone regeneration with the combination of particulate xenogeneic bone graft with no statistically significant differences in the primary measures of radiographic outcome across groups. The results confirm partial rejection of the null hypothesis, which suggests that the two membranes had similar bone regeneration, whereas PRF showed better soft tissue early healing. The amount of horizontal bone gain in the two groups (around 3.2 to 3.7 mm at the crestal level) can be considered within the range of values in the guided bone regeneration literature, in terms of the lateral ridge augmentation procedures. Past systematic review findings to have noted mean horizontal bone gains of 3.0 to 5.0 mm with resorbable collagen membrane together with particulate bone grafts [19], the current findings match these set standards. The fact that the collagen group recorded slight numerical superiority in the crestal horizontal bone gain but this was not statistically significant is worth consideration given the varying nature of biological behaviors of the two membranes. The cell-occlusive role of the barrier membranes is heavily reliant on the structural integrity of the membrane during the initial period of bone regeneration, which usually takes four to six months of continuous barrier functionalities [20]. Resorbable collagen membranes, especially cross-linked formulations are designed to preserve their structural and functional attributes between four-six months, hence enabling extended protection of the regenerated grafted space during the regenerative process [21. Conversely, PRF membranes are relatively biologically degraded and research has shown that there is a high rate of structural disappearance in two to four weeks [22]. This rapid breakdown curve is the major theoretical issue with using PRF as a barrier membrane on its own since a loss of barrier functionality may occur prematurely to allow soft tissue to penetrate the regenerating space before sufficient mineralization has taken place. Although this is not an ideal result of the theoretical approach, the similar overall bone regeneration results experienced in the PRF group indicate that there might be some compensatory biological processes that counteract the reduction in the barrier duration. The slow release of growth factors of the PRF fibrin matrix throughout the critical early healing period may boost osteoblast recruitment, proliferation and differentiation thus creating an early biological advantage compensating the decreased mechanical barrier period [23]. Also, the high concentration of fibrin in compressed PRF membranes can offer a sufficient cell occlusion during the first two to three weeks, which is the time frame of the highest proliferative activity of soft tissues [24]. Once this preliminary period is completed, the consolidating material in itself could be resistant to the intrusion of soft tissue by progressive mineralization and mixing with the native bone. The fact that the PRF group had a significantly higher score at day 7 in terms of the soft tissue healing index is a clinically significant finding. Early soft tissue healing is extremely important in guided bone regeneration surgeries since wound d ehiscence and membrane exposure are two of the most frequent complications which take place in up to 20% of managed by resorbable membranes [25]. It leads to barrier dysfunction and disease susceptibility, as well as a much lower bone regeneration rate in case of membrane exposure. The increased recovery rate of soft tissue with PRF is in line with the established biological activity of concentrated platelet-derived growth factors, especially platelet-derived growth factor and vascular endothelial growth factor that stimulate the growth of fibroblasts, extracellular matrix secretion and neoangiogenesis [26]. The biologically active wound dressing also can be the protective autologous fibrin layer that induces epithelial migration and decreases postoperative inflammation. The rate of wound dehiscence in the current study was less in PRF condition (5.3) than in collagen condition (16.7) though this was not statistically significant since the sample size was relatively small. Nevertheless, the clinical curve is in agreement with the past reports that PRF usage has decreased the complications of the wound in the bone augmentation surgeries [27]. To prove conclusively that the difference in dehiscence rates is a real treatment effect and not a chance accident, a bigger sample size would be required. The similarity in the vertical gain of the bones between the groups is interesting and indicates that neither membrane type had a serious advantage in vertical augmentation. Vertical bone regeneration is an intrinsically difficult task as compared to horizontal augmentation because of the need to maintain space against soft tissue collapses in the absence of bony walls (which support them) [28]. PRF and collagen membranes are both intrinsically soft and not stiff enough to maintain space independently, depending instead on the underlying particulate graft substance and tension on the wound to maintain the enhanced contour. This equal limitation can be one of the reasons why there was a similar value of vertical gain between the two groups. The gray value analysis of the bone density analysis through the CBCT procedure showed that there were similar values in the groups under study and that the quality of regenerated bone was similar no matter which type of membrane was used. The clinical implication of this finding is that bone density plays a direct role in determining primary implant stability and long-term findings of the outcome of the Secondary Osseointegration [29]. The similar density levels indicate that the growth factors produced by PRF did not show any significant improvements or deterioration to the mineralization of the regenerated tissue in the period of six months. Economically and practically, PRF has certain benefits compared to commercial collagen membranes made commercially. PRF is prepared by the use of simple blood collection and a single centrifugation step at minimum equipment costs and no commercial membrane products are required [30]. The total autologous content of PRF excludes the immunogenicity/disease spreading and ethical issues of animal collagen products, which can be crucial with regard to some groups of patients [31]. These practicability benefits, along with the similar regenerative efficacies exhibited in the current study, justify the inclusion of PRF in viable and economical alternative to collagen membranes in guided bone regeneration surgeries. This study has a number of limitations that should be mentioned. The sample size, though sufficient to provide evidence of large effect size, might have been too small to provide evidence of smaller but clinically significant group differences, especially in complication rates. The six months follow-up period only reflected the initial bone regeneration phase and it lacked the long-term stability of the bone that had been regenerated following the implant restoration under functional loading. Detailed information on bone quality, vital bone percentage and residual graft particle content would have been more accurately described by histomorphometric analysis using core biopsy specimens, which was not done out of ethical considerations, namely, with respect to further sampling of tissues [32]. Moreover, the experiment used one PRF preparation protocol and the difference in centrifugation parameters may have the potential impact on the biological characteristics and clinical functionality of the fibrin membrane [33]. Future conducting multicenter studies with increased sample sizes, extended follow-up, histological examination and cost-benefit evaluation would reinforce the evidence of clinical choice on the selection of membrane to be used in guided bone regeneration.

## Conclusion:

Both platelet-rich fibrin and collagen membranes are effective in promoting bone regeneration in alveolar defects.They demonstrate comparable outcomes in terms of bone gain and overall healing. Platelet-rich fibrin may offer additional benefits such as better soft tissue healing and cost-effectiveness due to its autologous nature

## Figures and Tables

**Table 1:** Demographic and baseline characteristics of study groups

**Parameter**	**PRF Group (n = 19)**	**Collagen Group (n = 18)**	**p-value**
Age (years), Mean ± SD	38.7 ± 9.4	40.2 ± 10.1	0.641
Sex (Male/Female)	8-Nov	9-Sep	0.598
Defect location (Maxilla/Mandible)	11-Aug	9-Sep	0.623
Baseline ridge width at crest (mm), Mean ± SD	3.42 ± 0.87	3.58 ± 0.93	0.589
Baseline vertical bone height (mm), Mean ± SD	9.14 ± 1.73	9.38 ± 1.86	0.683
Defect classification (Seibert I/III)	7-Dec	8-Oct	0.592
Time since extraction (months), Mean ± SD	8.3 ± 3.6	7.9 ± 4.1	0.752
*Statistically significant (p < 0.05)

**Table 2:** Clinical outcomes and soft tissue healing parameters

**Parameter**	**PRF Group (n = 19)**	**Collagen Group (n = 18)**	**p-value**
Healing index Day 7, Mean ± SD	4.21 ± 0.63	3.72 ± 0.75	0.028*
Healing index Day 14, Mean ± SD	4.74 ± 0.45	4.56 ± 0.51	0.256
Wound dehiscence, n (%)	1 (5.3%)	3 (16.7%)	0.334
Infection, n (%)	0 (0%)	1 (5.6%)	0.486
Excessive swelling (> 7 days), n (%)	2 (10.5%)	4 (22.2%)	0.4
Graft loss (partial/complete), n (%)	0 (0%)	1 (5.6%)	0.486

**Table 3:** Radiographic measurements and bone gain at six months

**Parameter**	**PRF Group (n = 19) Mean ± SD**	**Collagen Group (n = 18) Mean ± SD**	**p-value (between groups)**
**Horizontal bone width at crest (mm)**			
Baseline	3.42 ± 0.87	3.58 ± 0.93	0.589
6 months	6.63 ± 1.21	7.32 ± 1.34	0.104
Horizontal gain at crest	3.21 ± 1.06	3.74 ± 1.18	0.154
**Horizontal bone width at 3 mm (mm)**			
Baseline	4.18 ± 1.02	4.31 ± 0.98	0.692
6 months	7.86 ± 1.38	8.14 ± 1.42	0.539
Horizontal gain at 3 mm	3.68 ± 1.14	3.83 ± 1.27	0.702
**Horizontal bone width at 5 mm (mm)**			
Baseline	5.24 ± 1.16	5.41 ± 1.08	0.643
6 months	8.67 ± 1.52	8.93 ± 1.47	0.593
Horizontal gain at 5 mm	3.43 ± 1.22	3.52 ± 1.31	0.826
**Vertical bone height (mm)**			
Baseline	9.14 ± 1.73	9.38 ± 1.86	0.683
6 months	11.42 ± 1.89	11.67 ± 2.04	0.693
Vertical gain	2.28 ± 0.94	2.29 ± 1.08	0.974
Mean gray value (bone density)	684.3 ± 127.6	712.8 ± 134.2	0.503
